# Comparison Study of Different Extracts of *Plectranthus madagascariensis*, *P. neochilus* and the Rare *P. porcatus* (Lamiaceae): Chemical Characterization, Antioxidant, Antimicrobial and Cytotoxic Activities

**DOI:** 10.3390/biom9050179

**Published:** 2019-05-08

**Authors:** Diogo Matias, Marisa Nicolai, Ana Sofia Fernandes, Nuno Saraiva, Joana Almeida, Lucília Saraiva, Célia Faustino, Ana María Díaz-Lanza, Catarina P. Reis, Patrícia Rijo

**Affiliations:** 1 Research Center for Biosciences & Health Technologies (CBIOS), Universidade Lusófona de Humanidades e Tecnologias, Campo Grande 376, 1749-024 Lisboa, Portugal; diogohcmatias@gmail.com (D.M.); marisa.nicolai@ulusofona.pt (M.N.); ana.fernandes@ulusofona.pt (A.S.F.); nuno.saraiva@ulusofona.pt (N.S.); catarinareis@ff.ulisboa.pt (C.P.R.); 2 Department of Biomedical Sciences, Faculty of Pharmacy, University of Alcalá, Campus Universitario, 28871 Alcalá de Henares, Spain; ana.diaz@uah.es; 3 LAQV/REQUIMTE, Laboratório de Microbiologia, Departamento de Ciências Biológicas, Faculdade de Farmácia, Universidade do Porto, Rua de Jorge Viterbo Ferreira, 4050-313 Porto, Portugal; joanaalmeida15@gmail.com (J.A.); lucilia.saraiva@ff.up.pt (L.S.); 4 Research Institute for Medicines (iMed.ULisboa), Faculty of Pharmacy, Universidade de Lisboa, Av. Professor Gama Pinto, 1649-003 Lisboa, Portugal; cfaustino@ff.ulisboa.pt

**Keywords:** *Plectranthus*, Lamiaceae, diterpenes, antimicrobial, cytotoxicity

## Abstract

Medicinal plants of the *Plectranthus* genus (Lamiaceae) are known for their ethnopharmacological relevance, mainly against infectious, dermatologic and gastrointestinal pathologies. Three *Plectranthus* species originated from South Africa, namely *P. madagascariensis*, *P. neochilus* and the rare *P. porcatus* were hereby screened for their antimicrobial and cytotoxic activities related with their known and/or potential ethnomedicinal uses. Twenty-six extracts were prepared by the combination of extraction methods (infusion, decoction, microwave-assisted, ultrasound-assisted, maceration and supercritical fluid extraction) with different polarity solvents (water, methanol, acetone and supercritical CO_2_). The comparison study of these extracts was elucidated through the corresponding chemical characterization and cytotoxic activity data. Therefore, the acetone extract from *P. madagascariensis* prepared by ultrasound extraction method revealed potent antibacterial activity against Gram-positive bacteria (1.95 < minimum inhibitory concentration (MIC) < 7.81 μg/mL), including a methicillin-resistant *Staphylococcus aureus* (MRSA) strain. Additionally, acetone extracts from both *P. madagascariensis* and *P. neochilus* exhibited relevant antibacterial activity against Gram-negative *Klebsiella pneumonia* (0.48 < MIC < 3.91 μg/mL), validating the traditional uses of such plants as anti-infectious agents. All methanolic extracts showed potent antioxidant effects at 100 μg/mL measured as their radical scavenging activity (60.8–89.0%) in the 2,2-diphenyl-1-picrylhydrazyl (DPPH) assay. The *P. madagascariensis* extract obtained by maceration in acetone showed moderate cytotoxic effects in the MDA-MB-231 cell line (triple negative human breast carcinoma). The extract concentration that caused a 50% inhibition in cell viability (IC_50_) was 64.52 μg/mL. All extracts in this comparative study were profiled by high-performance liquid chromatography-HPLC with a diode-array detector-DAD (HPLC-DAD) and the main known bioactive components were identified in each extract, which included polyphenols (caffeic **1**, chlorogenic **2** and rosmarinic **3** acids), abietane diterpenes (7α-acetoxy-6β-hydroxyroyleanone **4** and coleon U **5**) and flavone glycosides (rutin **6** and naringin **7**).

## 1. Introduction

Plants have been used by humans since ancient times for medicinal purposes [1]. The herbal preparations are important healthcare resources remaining the most affordable medicines in developing countries [2]. The *Plectranthus* genus, comprising more than 300 species distributed through tropical and subtropical areas of Africa, Asia, Oceania and South America, belongs to the Lamiaceae family, which includes some well-established medicinal genus such as mint (*Mentha*), sage (*Salvia*) and thyme (*Thymus*). The *Plectranthus* species are commonly used as medicinal plants targeting infectious, dermatologic and gastrointestinal pathologies [3]. Their pharmacological properties have frequently been attributed to the presence of bioactive oxygenated diterpenes from the abietane, kaurane, phyllocladane, labdane, neoclerodane and halimane classes [4]. In this study, three *Plectranthus* species were selected based on their known and/or potential ethnomedicinal uses, namely *P. madagascariensis, P. neochilus* and the rare *P. porcatus*. These aqueous *Plectranthus* extracts have already been described and are herein compared to the organic and supercritical fluid extracts. The chemical composition and biological activity will also be discussed and compared. Different extraction methodologies (decoction, infusion, maceration, microwave-assisted, ultrasound-assisted and supercritical fluid extractions) using several extraction solvents (water, acetone, methanol and supercritical CO_2_) were employed. The comparison of all solvents and extraction techniques was analyzed to uncover the efficiencies for each compound extracted. Moreover, the correlation study of the antioxidant, antimicrobial and cytotoxic activities of each extract obtained with the corresponding phytochemical composition was also achieved. Antioxidant activity of *Plectranthus* extracts was complementary assayed since the therapeutic effect of medicinal plants has frequently been associated with the presence of antioxidant constituents, like phenolic compounds. These can offer protection against radical oxygen species (ROS) damage involved in many pathological conditions, including cancer, although in vivo effects depend on the compound absorption and bioavailability [5].

**Figure 1:**
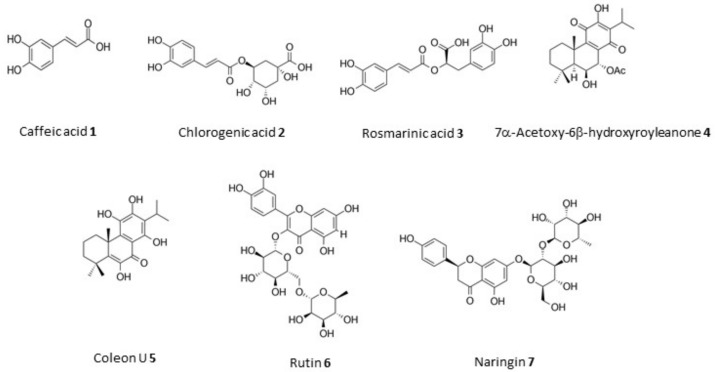
Structures of caffeic **1**, chlorogenic **2** and rosmarinic **3** acids, abietane diterpenes (7α-acetoxy-6β-hydroxyroyleanone **4** and coleon U **5**) and flavone glycosides (rutin **6** and naringin **7**).

*Plectranthus madagascariensis* (Pers.) Benth. is a perennial aromatic herb [6] traditionally used for the treatment of respiratory conditions, such as cough and asthma, cutaneous wounds and scabies [3]. The *P. madagascariensis* essential oil has been previously found to contain high yields of the abietane diterpene 6α,7β-dehydroroyleanone [7]. This diterpenoid was characterized as a non-toxic weak antimicrobial agent and a potent antioxidant compound [8]. An acetone extract of *P. madagascariensis* has shown antimicrobial and insect antifeedant activities which were attributed to the presence of another abietane diterpene, coleon U **5** (Figure 1) [9]. An aqueous extract of *P. madagascariensis* rich in rosmarinic acid **3** showed relevant activity against *Staphylococcus epidermidis* and has been incorporated into a polymeric alginate formulation to improve long-term stability, being the first description of the potential use of this plant to treat skin pathologies [10]. Later, Kubínová et al. [11] identified rosmarinic acid **3**, 6β,7β-dihydroxyroyleanone, 7β-acetoxy-6β-hydroxyroyleanone **4** (Figure 1) and coleon U quinone as the main components of a methanolic extract of *P. madagascariensis*. The abietane royleanones isolated from this extract exhibited potent antibacterial activity against *Staphylococcus aureus* and *Enterococcus faecalis* [11].

*Plectranthus neochilus* Schltr. is an aromatic herb known as “boldo-rasteiro” in Brazil [12]. The infusion of this plant has been used in traditional medicine for the treatment of dyspepsia and hepatic insufficiency [12] and also for the treatment of chills, cough and a runny or blocked nose [13]. Several studies characterizing the essential oil composition and respective biologic activities of plants of different growth locations have been reported. The compositions of essential oils from plants grown in Portugal [14] and Brazil [15] were similar, however, those were substantially different from plants grown in South Africa [16]. Monoterpene hydrocarbons were the most prevalent volatile constituents, but sesquiterpenes such as β-caryophyllene and its oxidized form, caryophyllene oxide, were also present in high yields [16]. The antimicrobial [14,17], schistosomicidal [15,18], anti-fungal [19] and cytotoxic [15,19] activities were evaluated for the *P. neochilus* essential oils. A low toxicity for the *P. neochilus* ethanol extract of the aerial parts was also described based on the brine shrimp lethality assay [20].

*Plectranthus porcatus* Van Jaarsv. & P.J.D. Winter is a highly aromatic plant endemic from the Limpopo province in South Africa, described for the first time in 2004 [21], and its potential medicinal properties are poorly understood. This plant has been briefly studied by our group and a new diterpene with a cycloabietane substructure, (13*S*,15*S*)-6β,7α,12α,19-tetrahydroxy-13β,16-cyclo-8-abietene-11,14-dione, was obtained from the acetone extract [22], which showed antibacterial activity (62.5 < minimum inhibitory concentration (MIC) < 125 μg/mL) against Gram-positive microorganisms [22]. The microwave aqueous extract was analyzed by high-performance liquid chromatography (HPLC) and small yields of the polyphenols **1** and **3** (Figure 1) were detected, which contributed to the medium antioxidant activity of this extract [23]. Thus, *P. porcatus* was included in this study in order to further elucidate its biologic properties and potential medicinal applications since information regarding its uses and current exploitation are lacking.

## 2. Materials and Methods

### 2.1. Cell Lines, Chemicals and Biochemicals

Extraction solvents (*n*-hexane, ethyl acetate, methanol and acetone) were from analytic grade and purchased to Sigma-Aldrich (Steinheim, Germany). Reverse osmosis water with a resistivity of 18.2 Ω⋅cm at 25 °C was obtained from a Millipore system (Millipore, Burlington, MA, USA). Trichloroacetic acid was from Panreac (Barcelona, Spain). HPLC solvents of HPLC-grade were obtained from VWR Chemicals (Fontenay-sous-Bois, France) and were further filtered through a 0.22 μm membrane (Vygon, Ecouen, France) and degassed before use. Dimethyl sulfoxide (DMSO) and absolute ethanol were from Merck (Darmstadt, Germany). Ascorbic acid, 2,2-diphenyl-1-picrylhydrazyl (DPPH), caffeic acid, chlorogenic acid, rosmarinic acid, rutin, naringin, vancomycin, norfloxacin, amphotericin B and doxorubicin were from Sigma-Aldrich. Authentic standards of coleon U and 7α-acetoxy-6β-hydroxyroyleanone were obtained and fully characterized by Gaspar-Marques [24]. Mueller-Hinton broth was supplied by Sigma-Aldrich and Sabouraud agar was supplied by Biokar Diagnostics (Allone, France). Fetal bovine serum and penicillin/streptomycin for cell culture were from Sigma-Aldrich while Dulbecco’s Modified Eagle’s medium (DMEM) was from Biowest (Nuaillé, France). The human breast cancer MDA-MB-231 cell line was obtained from ATCC and maintained at CBIOS at Universidade Lusófona de Humanidades e Tecnologias (Lisbon, Portugal) facilities. Previously to the biologic tests, samples were dissolved in DMSO or ultrapure water in the case of water-soluble samples.

### 2.2. Plant Material

*Plectranthus madagascarensis* Benth., *P. neochilus* Schltr. and *P. porcatus* Winter & Van Jaarsv were cultivated in Parque Botânico da Tapada da Ajuda (Instituto Superior Agrário, Lisbon, Portugal) from cuttings obtained from the Kirstenbosch National Botanical Garden (Cape Town, South Africa). Voucher specimens of *P. madagascariensis* (841/2007), *P. neochilus* (570/2008) and *P. porcatus* (109/2008) were deposited in the Herbarium João de Carvalho e Vasconcellos (Instituto Superior Agrário, Lisbon, Portugal). The plant material was collected between 2007 and 2008, dried at room temperature and stored protected from light and humidity.

### 2.3. Extract Preparation

Dried plants were ground to small pieces and then pulverized. The plant material was extracted using different combinations of solvents (water, methanol, acetone and supercritical CO_2_) and extraction techniques. Each crude extract was separated from the remaining plant material by paper filtration (Whatman paper #1, Sigma-Aldrich) and the organic solvents were evaporated in a rotary evaporator (IKA RV06-ML 1-B, Staufen, Germany) bellow 40 °C while aqueous extracts were freeze-dried as 1 mL aliquots (Freezone 2.5 L, Labconco, Kansas City, USA). Each dried extract was weighted and stored at −20 °C until further use.

The aqueous extracts were prepared by decoction (DEC), infusion (INF) or microwave-assisted extraction (MW). In the first procedure (DEC), the plant material (10 g) was boiled in 100 mL of distilled water for 10 min while in the latter procedures the plant material (10 g) was added to 100 mL of boiling distilled water and kept in contact with it for 10 min (INF) or alternatively added to 100 mL of distilled water subject to continuous irradiation (2.45 GHz) for 2 min (MW) in a conventional microwave oven. Organic extracts were prepared by maceration (MA) or ultrasound-assisted extraction (US) by adding the plant material (10 g) to 200 mL of organic solvent (acetone or methanol) kept stirring for 24 h (MA) or sonicated in an ultrasonic bath (Bandelin SONOREX RK 510H, Berlin, Germany) at 35 kHz for 2 h (US). Alternatively, supercritical fluid extraction (SCFE) was also performed by packing the plant material (30 g) into a 278 cm^3^ inox cell and injecting supercritical carbon dioxide (scCO_2_) during 3 h at a flow rate of 0.3 kg/h under a pressure of 230 bar and temperature of 40 °C [25]. The remaining plant material resulting from SCFE was recovered, air dried and re-extracted (R-SCFE) by addition to 200 mL of acetone which was kept stirring for 24 h at room temperature.

### 2.4. DPPH Radical Scavenging Assay

The antioxidant activity was screened by evaluation of the DPPH radical scavenging ability [23]. Briefly, 10 μL of each plant extract (10 mg/mL) were mixed with 990 μL of DPPH solution (0.002% in ethanol). After 30 min incubation at room temperature, absorbance was measured at 517 nm against a corresponding blank. The antioxidant activity (AA, %) was calculated as:

AA (%) = (*A*_DPPH_ − *A*_sample_)/*A*_DPPH,_ (1)

where *A*_DPPH_ is the absorbance of DPPH solution (in the absence of sample extract) and *A*_sample_ is the absorption of sample solution. Assays were carried out in triplicate and ascorbic acid was used as positive control.

### 2.5. Antimicrobial Activity Assays

The antimicrobial activity of *Plectranthus* spp. extracts was determined in vitro against Gram-positive bacteria strains *Bacillus subtilis* (ATCC 6633), *Enterococcus faecalis* (ATCC 29212), *Mycobacterium smegmatis* (ATCC 607), *Staphylococcus aureus* (ATCC 25923), methicillin-resistant *S. aureus* (CIP 106760) and *S. epidermidis* (ATCC 12228), Gram-negative bacteria strains *Escherichia coli* (ATCC 25922), *Klebsiella pneumoniae* (ATCC 9997) and *Pseudomonas aeruginosa* (ATCC 27853), and the yeasts *Candida albicans* (ATCC 10231) and *Saccharomyces cerevisiae* (ATCC 9763). Microbial strains were originally obtained from American Type Culture Collection (ATCC) or form “Collection de l’Institut Pasteur” (CIP).

The well diffusion assay was used for screening of the antimicrobial activity. Briefly, 100 µL of microorganism suspension, concentrated at 0.5 in McFarland scale, was inoculated in a Petri dish containing Mueller-Hinton (bacteria) or Sabouraud agar (yeasts). Wells were dug in the agar using a sterile Pasteur pipette. Then 50 µL of each sample (10 mg/mL), negative control (DMSO) or positive control (vancomycin for Gram-positive bacteria, rifampicin for mycobacteria, norfloxacin for Gram-negative bacteria or amphotericin B for yeasts) were added to each well. After incubation at 37 °C for 24 h the growth inhibition zones around the wells were measured and the results were expressed in millimetres (mm) as median values of at least three replicates [10].

Positive samples (capable of inhibiting microbial growth) from the well diffusion assay screening were subjected to a microplate broth microdilution method [26] in order to determine the minimum inhibitory concentration (MIC) values. Briefly, 100 μL of liquid Mueller-Hinton medium was distributed in each well of a 96-well plate. To the first well of each row were added 100 μL of each extract, positive control or negative control solutions at 1 mg/mL concentration and 1:2 serial dilutions were prepared (1.95–500 μg/mL range). Lastly, 10 μL of bacterial suspension were added to all wells and plates were incubated at 37 °C for 24 h. Bacterial growth was evaluated with an absorbance microplate reader (Thermo Scientific Multiskan FC, Loughborough, UK) set at 620 nm. Data was expressed as the median values of at least three replicates.

### 2.6. Cytotoxicity Evaluation

The cytotoxicity of *Plectranthus* spp. extracts was assessed in the human breast cancer MDA-MB-231 cell line [27]. Cells were cultured in DMEM supplemented with 10% fetal bovine serum, 100 U/mL penicillin and 0.1 mg/mL streptomycin. The cultures were maintained at 37 °C under a humidified atmosphere containing 5% CO_2_. Cell viability was evaluated using the crystal violet staining assay [27]. Briefly, a 96-well microplate was inoculated with approx. 6000 cells per well and incubated for 24 h. The samples were then added to obtain a final concentration of 15 µg/mL. After 48 h the medium was discarded and the cells were washed with phosphate buffer saline (PBS), fixed with 96% ethanol and stained with crystal violet. The absorbance was measured at 595 nm and the sample cytotoxicity was expressed as the fraction of absorbance comparing with non-treated control cultures. At least two independent experiments were performed, and four replicate cultures were used in each independent experiment. Doxorubicin (5 µM) was used as positive control.

### 2.7. HPLC-DAD Fingerprinting

Chemical characterization of plant extracts, namely their phytochemical profile, was obtained by high-performance liquid chromatography with diode array detection (HPLC-DAD) analysis performed with an Agilent Technologies 1200 Infinity Series LC system (Agilent Technologies, Santa Clara, CA, USA) coupled to a diode array detector (DAD). A 20 μL sample extract (10 mg/mL) was injected into a reverse phase LiChrospher^®^ 100 RP-18 5 μm (4.0 × 250 mm) column (Merck) and eluted with a gradient composed of methanol (A), acetonitrile (B) and 0.3% trichloroacetic acid in water (C) as follows: 0 min, 15% A, 5% B and 80% C; 20 min, 80% A, 10% B and 10% C and 25 min, 80% A, 10% B and 10% C. The flow rate was set at 1 mL/min at room temperature. Detection was carried out between 200 and 600 nm. All analyses were performed in triplicate.

Additionally, the main components of the extracts were identified by pure standards overlay of ultra violet (UV) spectra and retention time [28]. Standards (1 mg/mL) in methanol were run by injecting 20 µL sample under the same analytical conditions used for the extracts. The UV spectra of each pure compound was retrieved and compared to peaks in the extract with similar retention times. When overlay of UV spectra was observed, a co-elution of the extract with the pure compound was performed for confirmation.

### 2.8. Statistical Analysis and Software Editing

Statistical analysis and graphic design were archived using GraphPad Prism 6.01 (San Diego, California, USA) for Windows 10. ChemStation (Santa Clara, CA, USA) was used for HPLC-DAD controller and data exportation. Chemical structures were drawn on ChemBioDraw Ultra 12.0.2.1076 (Luton Bedfordshire, UK).

## 3. Results and Discussion

Several extracts from *P. madagascariensis*, *P. neochilus* and *P. porcatus* were prepared with different solvents (water, acetone, methanol and supercritical CO_2_) and extraction techniques (decoction, infusion, maceration, microwave-assisted, ultrasound-assisted and supercritical fluid extractions) to achieve preferential extraction of dissimilar polarity constituents obtained in variable yields (Table 1). To the best of our knowledge, this is the first comparison study regarding the preparation of different extracts using different organic and aqueous solvents from these three *Plectranthus* spp. Higher extraction yields were generally obtained by the maceration method and using methanol as a solvent which can be due to the ability of the alcohol to disrupt the plant cell wall with higher efficacy compared to the other solvents employed. The antioxidant activity of the extracts was also determined based on their radical scavenging ability (Table 1) towards the free radical 2,2-diphenyl-1-picrylhydrazyl (DPPH) since the presence of antioxidant constituents in the *Plectranthus* extracts can contribute to attenuate oxidative stress and prevent free radicals from damaging biomolecules such as membrane lipids, proteins and DNA [5].

Antioxidant activity was higher in the methanol extracts (Table 1) in comparison with the extracts obtained using the remaining solvents, but the antioxidant activity of the methanolic extracts is slightly lower than the one of the ascorbic acid, used as positive control in the DPPH assay. Moreover, the most active were the ones obtained from *P. madagascariensis* (E6 and E7), *P. neochilus* (E15 and E16) and *P. porcatus* (E23 and E24). The aqueous extracts of *P. neochilus* also showed relevant antioxidant capacity (62.5–68.9%) in contrast to aqueous extracts of *P. madagascariensis* (10.2–20.6%) and *P. porcatus* (3.2–17.3%). Aqueous extracts of *P. neochilus* and *P. porcatus* were previously described and are in agreement with literature results [23].

The high antioxidant activity of the methanolic extracts can be attributed to the presence of higher amounts of polyphenols (latter identified by HPLC-DAD profiling) in accordance with previous studies of *Plectranthus* plants that revealed the extraction of high amounts of such compounds when using methanol as solvent [9,11,29].

The screening of antimicrobial activity of all *Plectranthus* spp. extracts was initially assessed in vitro against five Gram-positive microorganisms (*Bacillus subtilis*, *Enterococcus faecalis*, *Staphylococcus aureus*, *S. epidermidis*, and *Mycobacterium smegmatis*), three Gram-negative bacteria (*Escherichia coli*, *K. pneumoniae*, and *Pseudomonas aeruginosa*) and two yeasts (*Candida albicans* and *Saccharomyces cerevisiae*) using the well diffusion assay. No microbial growth inhibitory activity was observed for *E. faecalis*, *E. coli*, *P. aeruginosa* or the two yeast strains by any of the extracts tested, which is in accordance with previous works [10]. Moreover, *P. porcatus* extracts were inactive against all the microbial strains assessed. Results for the bioactive extracts (Table 2) show that both the acetonic and methanolic extracts of *P. madagascariensis* (E4–E7) and *P. neochilus* (E13–E16) obtained either by maceration or ultrasound-assisted extraction were active against most of the Gram-positive microorganisms, including mycobacteria. On the other hand, only the acetone extracts of *P. madagascariensis* (E4 and E5) were able to inhibit Gram-negative bacteria (*K. pneumoniae*). Extracts (Table 1) not shown in Table 2 were inactive against all the microbial strains tested.

Pursuing the antimicrobial studies, the MIC values for the three most active *Plectranthus* extracts (E4, E5, and E13) identified in the well-diffusion assay screening (Table 2), which were all acetone extracts obtained by maceration and/or ultrasound-assisted extraction from *P. madagascariensis* and *P. neochilus*, were determined in the susceptible strains. These extracts showed potent antibacterial activity, namely against Gram-negative *K. pneumoniae*, with MIC values between 0.48 and 3.91 μg/mL (Table 3). The acetone extract from *P. madagascariensis* (E4) prepared by the ultrasound-assisted extraction method showed the most potent activity against Gram-positive bacteria, including a methicillin-resistant *S. aureus* (MRSA) strain, with MIC values ranging from 1.95 to 7.81 μg/mL comparable to reference antibiotics. The inhibitory effect obtained from the bioactive extracts is mainly due to the diterpenoids extracted by the corresponding apolar extraction solvents (Table 2 and Table 3). This is in agreement with previous *Plectranthus* spp. studies which attributed the bioactivity to the bioactive royleanone diterpenoids obtained from nonpolar extraction solvent, or rosmarinic acid from polar extraction solvent [10,11,22,23,24,25,26].

**Figure 2:**
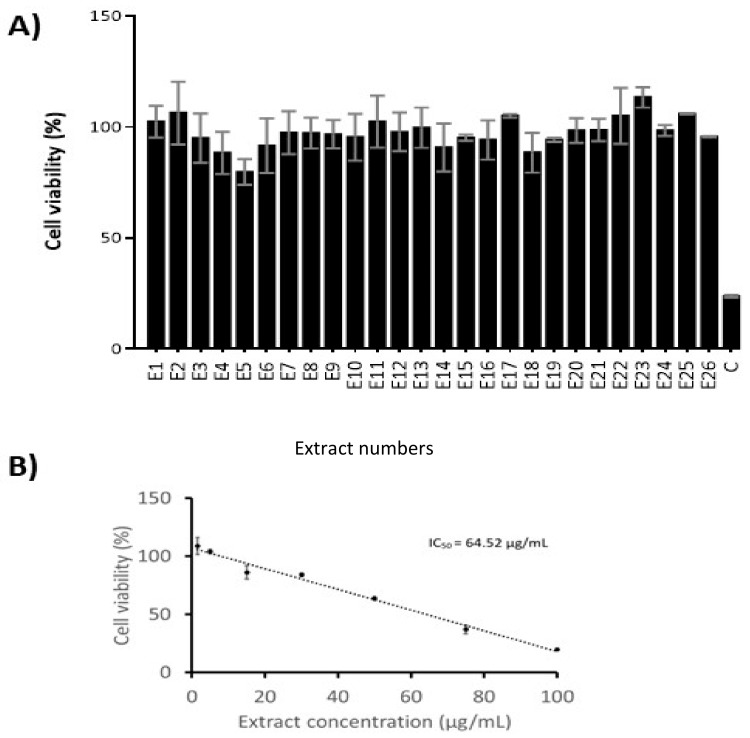
(**A**) The viability of MDA-MB-231 cells exposed to *Plectranthus* spp. extracts (15 μg/mL) for 48 h assessed by the crystal violet staining assay using doxorubicin (DOX, 5 μM) as positive control; (**B**) Concentration-response profile for the *P. madagascariensis* extract obtained by maceration in acetone. Results are expressed as mean ± standard deviation (SD) from at least two independent experiments. IC_50_: extract concentration producing 50% inhibition of cell viability.

The cytotoxicity of the *Plectranthus* extracts was evaluated in MDA-MB-231 breast cancer cells selected due to their availability, ease of manipulation and lack of studies of *Plectranthus* plant extracts in this cell line. All extracts at a concentration of 15 μg/mL showed low cytotoxicity towards MDA-MB-231 breast cancer cells (Figure 2A). The most cytotoxic extract was the one obtained by maceration in acetone of *P. madagascariensis* (E5), with a reduction in cell viability of 20.13%, followed by the extract (E18) obtained by re-extraction in acetone of *P. neochilus* plant material previously extracted by SCFE (11.44% decrease in cell viability). The extract concentration producing 50% inhibition of cell viability (IC_50_) was determined for the most active extract (E5) and a value of 64.52 μg/mL was found (Figure 2B). This extract can thus be considered moderately cytotoxic [29] and was selected for further studies in other cancer cell lines, which are currently ongoing. 

**Figure 3:**
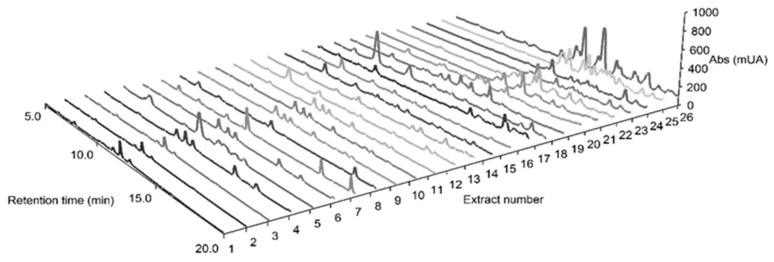
High-performance liquid chromatography (HPLC) fingerprinting (5–20 min section) of *Plectranthus* spp. extracts (10 mg/mL) obtained at 270 nm, chemical structures of their predominant constituents and correspondent retention times (Rt, min). Abs: absorbance.

The phytochemical composition of the crude extracts was analyzed by HPLC-DAD in order to profile the most prevalent components (Figure 3), which were additionally identified by standard co-elution. Although the constituents of *P. madagascariensis* aqueous [23], acetonic [9] and methanolic [11] extracts have been reported, to the best of our knowledge there is no published HPLC-DAD data on *P. neochilus* or *P. porcatus* extracts. The detection was carried out between 200 and 600 nm and the results are shown in Figure 3 at 270 nm.

For a better understanding of Figure 3, the observation of Table 4 compares the compounds detection in each *Plectranthus* spp. extract with their corresponding characteristic retention times.

The HPLC-DAD fingerprinting of *P. madagascariensis* extracts confirmed the presence of some known compounds identified in previous studies [9,11,23], namely the phenolic compounds **1**, **2** and **3**, which were present in all of these plant extracts (E1–E9), and the abietane diterpenes 7α-acetoxy-6β-hydroxyroyleanone **4** and coleon U **5** (Figure 3), common in the organic extracts obtained using the less polar solvents acetone (E4–E5) and supercritical CO_2_ (E8–E9). Thus, the higher antimicrobial and cytotoxic activity of the acetonic extracts of *P. madagascariensis* can be attributed to the presence of these abietane diterpenes, which have been previously isolated from other *Plectranthus* species and shown to possess potent antibacterial [29,30] and antitumoral [31] activities, being identified as antiproliferative agents against some cancer cell lines [32,33]. The presence of those compounds could also explain the traditional use of this plant for the treatment of respiratory conditions related to infectious agents and it is also consistent with the use of *P. madagascariensis* for the treatment of cutaneous wounds and scabies [3].

Coleon U **5** was also characterized as a protein kinase C (PKC)-δ activator which can contribute to its cytotoxic activity [34] and the presence of this diterpene can be responsible for the most relevant cytotoxic effects obtained for the *P. madagascariensis* extract (E5) prepared by maceration in acetone. The highest antioxidant activity comparable to that of positive control ascorbic acid was also obtained for this plant methanolic extract (E6) prepared by ultrasound-assisted extraction. Since high yields of polyphenols are also present in the extract, the concomitant radical scavenging activities of both abietane diterpenes and polyphenols could explain this relevant antioxidant activity.

The flavonoid glycosides rutin and naringin were also detected in the studied *Plectranthus* spp. extracts. Rutin was identified for the first time in these plant extracts being found in both organic and aqueous extracts, including the aqueous and/or acetonic extracts of *P. madagascariensis* (E1–E4), *P. neochilus* (E10–E15) and *P. porcatus* (E19–E20). Naringin found in *P. porcatus* extracts (E19–E22, E24, and E26) was detected for the first time in the *Plectranthus* genus.

The extracts from *P. neochilus* (E10–E18) were rich in polyphenols, including acids **1**, **2** and **3**, but the flavonoid glycoside **6** was also present both in the aqueous extracts (E10–E12) and inaf the organic ones prepared by ultrasound-assisted extraction using either acetone or methanol as solvent (E13 and E15). Polyphenols were also found in most of the *P. porcatus* extracts (chlorogenic acid **2** in E20, E22, E25, and E26; caffeic acid **1** in E19–E22, E24, and E26; rosmarinic acid **3** in E19 and E26) while flavonoid glycosides rutin **6** and naringin **7** were present in all aqueous extracts of this plant. Many of those compounds have been shown to possess both antimicrobial [35] and antioxidant [23] activities in previous studies, thus contributing to the observed activities in such plant extracts. Although *P. neochilus* and *P. porcatus* extracts did not show activities as potent as those of *P. madagascariensis* despite their content rich in polyphenols and some flavonoids, the interesting antimicrobial activity of the acetonic extract obtained from *P. neochilus* (E13) by the ultrasound technique could explain the traditional use of this plant for the treatment of infection-related symptoms such as cough or chills [13].

In conclusion, the preparation and bioactivity screening of different extracts from the three *Plectranthus* species studied led to the identification of extracts with antimicrobial, antioxidant and cytotoxic activities. Thus, the properties of each extract can be attributed to the presence of polyphenolic acids **1**, **2** and **3** (caffeic, chlorogenic and rosmarinic acids) present in most of the extracts along with flavonoid glycosides (rutin **6** and naringin **7**) or abietane diterpenes (7α-acetoxy-6β-hydroxyroyleanone **4** and coleon U **5**) detected by HPLC-DAD analysis. The methanol extracts were the ones with higher yield, so those with higher content in polar compounds, namely rosmarinic acid **3**. The aqueous extracts do not obtain diterpenoids resulting in no cytotoxic extracts. In addition, the more polar extracts have more antioxidant activity resultant from the phenolic compounds. The acetonic extracts are the ones with a high amount of diterpenes and so those with antimicrobial activity and potent cytotoxic activity. In general, to achieve bioactive *Plectranthus* extracts attributed to the cytotoxicity, ultrasound-assisted extractions using a low quantity of solvent and rapid efficient extractions should be performed. This comparative study thus contributed to the characterization of bioactivities and phytochemical composition of three *Plectranthus* species and to the scientific validation of the bioactive *P. madagascariensis* and *P. neochilus* ethnomedicinal uses. Additionally, these results highlight the potential use of *P. porcatus* as a medicinal plant in the future despite its rarity, remoteness and strong smell [21].

## Figures and Tables

**Table 1:** *Plectranthus* spp. extraction yields according to solvent and extraction method, and radical scavenging activity (RSA) of the extracts (100 μg/mL) in the DPPH assay.

Plant	Solvent	Extraction Method ^a^	Dry Residue (g)	Yield (mg/g)	Extract	RSA ^b^ (%)
*Plectranthus madagascariensis*	Water	INF	0.23 ± 0.10	2.3	E1	<50
		MW	0.11 ± 0.04	1.1	E2	<50
		DEC	0.22 ± 0.02	2.2	E3	<50
	**Acetone**	US	0.151	1.5	E4	<50
		**MA**	0.377	**3.8**	E5	<50
	**Methanol**	**US**	0.656	**6.6**	**E6**	**89.0**
		**MA**	1.146	**11.5**	**E7**	**64.8**
	scCO_2_	SCFE	0.394	0.01	E8	<50
	Acetone	R-SCFE	0.885	0.03	E9	<50
*Plectranthus neochilus*	Water	INF	0.26 ± 0.04	2.6	E10	<50
		MW	0.15 ± 0.04	1.5	E11	<50
		DEC	0.22 ± 0.01	2.2	E12	<50
	Acetone	US	0.180	1.8	E13	<50
		MA	0.125	1.3	E14	<50
	**Methanol**	**US**	0.702	**7.0**	**E15**	**64.9**
		**MA**	0.600	**6.0**	**E16**	**62.3**
	scCO_2_	SCFE	0.251	0.8	E17	<50
	Acetone	R-SCFE	0.417	1.4	E18	<50
*Plectranthus porcatus*	Water	INF	n/d	n/d	E19	<50
		MW	n/d	n/d	E20	<50
	Acetone	US	0.865	8.7	E21	<50
		MA	0.872	8.7	E22	<50
	**Methanol**	**US**	1.566	**15.7**	**E23**	**60.8**
		**MA**	2.237	**22.4**	**E24**	**65.9**
	scCO_2_	SCFE	0.191	0.64	E25	<50
	Acetone	R-SCFE	0.868	2.9	E26	<50

^a^ DEC, decoction; INF, infusion; MA, maceration; MW, microwave-assisted extraction; SCFE, supercritical fluid extraction; R-SCFE, re-extraction of the SCFE remaining plant material; US, ultrasound-assisted extraction; n/d, not determined. ^b^ Positive control (ascorbic acid): 93.4%. In bold the most relevant results. DPPH: 2,2-diphenyl-1-picrylhydrazyl.

**Table 2:** Diameter (mm) of microbial growth inhibition by bioactive extracts (10 mg/mL) in the well diffusion assay.

Microbial Strains	Extract								Positive Control ^a^
	E4	E5	E6	E7	E13	E14	E15	E16	
**Gram-positive**									
*Bacillus subtilis* ATCC 6633	**24**	**20**	12	11	15	14	11	10	31 (VAN)
*Staphylococcus. aureus* ATCC 25923	**24**	**20**	7	8	8	8	8	8	24 (VAN)
*Staphylococcus. epidermidis* ATCC 12228	10	**25**	nt	13	15	11	nt	5	20 (VAN)
*Mycobacterium smegmatis* ATCC 607	**26**	**26**	23	17	20	15	15	20	33 (RIF)
**Gram-negative**									
*Klebsiella. pneumoniae* ATCC 9997	**25**	**22**	5	5	5	5	5	5	25 (NOR)

^a^ VAN, vancomycin (Gram-positive); RIF, rifampicin (mycobacteria) or NOR, norfloxacin (Gram-negative); well diameter, 5 mm; nt, not tested. In bold the most relevant results.

**Table 3:** Minimum inhibitory concentration (MIC) values (μg/mL) for the most active *Plectranthus* spp. extracts.

Microbial Strains	Extract			Positive Control ^a^
	E4	E5	E13	
**Gram-positive**				
*B. subtilis* ATCC 6633	**3.91**	62.5	125	<0.48 (VAN)
*S. aureus* ATCC 25923	**3.91**	250	250	7.81 (VAN)
*S. aureus* CIP 106760	**1.95**	15.62	31.25	<0.98 (VAN)
*S. epidermidis* ATCC 12228	**7.81**	62.5	62.5	7.81 (VAN)
*M. smegmatis* ATCC 607	31.25	62.5	15.62	<0.48 (RIF)
**Gram-negative**				
*Klebsiella pneumoniae* ATCC 9997	**<0.48**	**3.91**	**0.98**	15.62 (NOR)

^a^ Vancomycin (Gram-positive), rifampicin (mycobacteria) or norfloxacin (Gram-negative); well diameter, 5 mm. In bold the most relevant results.

**Table 4:** Compounds detection in each *Plectranthus* spp. extract and their corresponding characteristic retention times.

Extract *n*	RT (min)						
	Clo 6.54	Caf 8.32	Nar 10.14	Rut 11.67	Ros 12.43	Roy 14.75	ColU15.44
E1	+	+	−	+	+	−	−
E2	+	+	−	+	+	−	−
E3	+	+	−	+	+	−	−
E4	+	+	−	+	+	+	−
E5	+	+	−	−	+	+	+
E6	+	+	−	−	+	−	−
E7	+	+	−	−	+	−	−
E8	+	+	−	−	+	+	−
E9	+	+	−	+	+	+	−
E10	+	+	−	+	+	−	−
E11	+	+	−	+	+	−	−
E12	+	+	−	+	+	−	−
E13	+	+	−	+	+	−	−
E14	+	+	−	−	+	−	−
E15	+	+	−	+	+	−	−
E16	+	+	−	−	+	−	−
E17	+	−	−	−	−	−	−
E18	+	+	−	−	+	−	−
E19	−	+	+	+	+	−	−
E20	+	+	+	+	+	−	−
E21	−	+	+	−	−	−	−
E22	+	+	+	−	+	−	−
E23	−	−	−	−	−	−	−
E24	−	+	+	−	−	−	−
E25	+	−	−	−	−	−	−
E26	+	+	+	−	+	−	−

RT, characteristic retention time in the developed HPLC method; Clo, Chlorogenic acid **2**; Caf, Cafeic acid **1**; Nar, Naringin **7**; Rut, Rutin **6**; Ros, Rosmarinic acid **3**; Roy, 7α-acetoxy-6β-hydroxyrooyleanone **4**; ColU, Coleon U **5**. + detected compound; − not detected.
